# Calibration and assessment of channel-specific biases in microarray data with extended dynamical range

**DOI:** 10.1186/1471-2105-5-177

**Published:** 2004-11-12

**Authors:** Henrik Bengtsson, Göran Jönsson, Johan Vallon-Christersson

**Affiliations:** 1 Mathematical Statistics, Centre for Mathematical Sciences, Lund University, Box 118, SE-221 00 Lund, Sweden; 2 Department of Oncology, Lund University, Barngatan 2, SE-221 85 Lund, Sweden

## Abstract

**Background:**

Non-linearities in observed log-ratios of gene expressions, also known as intensity dependent log-ratios, can often be accounted for by global biases in the two channels being compared. Any step in a microarray process may introduce such offsets and in this article we study the biases introduced by the microarray scanner and the image analysis software.

**Results:**

By scanning the same spotted oligonucleotide microarray at different photomultiplier tube (PMT) gains, we have identified a channel-specific bias present in two-channel microarray data. For the scanners analyzed it was in the range of 15–25 (out of 65,535). The observed bias was very stable between subsequent scans of the same array although the PMT gain was greatly adjusted. This indicates that the bias does not originate from a step preceding the scanner detector parts. The bias varies slightly between arrays. When comparing estimates based on data from the same array, but from different scanners, we have found that different scanners introduce different amounts of bias. So do various image analysis methods. We propose a scanning protocol and a constrained affine model that allows us to identify and estimate the bias in each channel. Backward transformation removes the bias and brings the channels to the same scale. The result is that systematic effects such as intensity dependent log-ratios are removed, but also that signal densities become much more similar. The average scan, which has a larger dynamical range and greater signal-to-noise ratio than individual scans, can then be obtained.

**Conclusions:**

The study shows that microarray scanners may introduce a significant bias in each channel. Such biases have to be calibrated for, otherwise systematic effects such as intensity dependent log-ratios will be observed. The proposed scanning protocol and calibration method is simple to use and is useful for evaluating scanner biases or for obtaining calibrated measurements with extended dynamical range and better precision. The cross-platform R package aroma, which implements all described methods, is available for free from .

## Background

The microarray technology provides a way of simultaneously measuring transcript abundances of 10^3 ^– 10^5 ^genes from one or more cell or tissue samples. A microarray, also known as a gene chip, has well defined regions that each consists of immobilized sequences of DNA, which each is unique to a specific gene. These regions are referred to as *probes *[1]. When fluorophore labeled cDNA, referred to as *targets*, obtained by reverse transcription of mRNA extracted from the samples of interest is let to hybridize to the probes for a few hours, each region on the microarray will specifically bind a certain amount of hybridized DNA unique to the corresponding gene. Depending on if a two-channel or single-channel microarray platform is used, either several and differentially labeled targets are hybridized to the same array, or different targets are each hybridized to separate arrays using identical labels. Next, the array is scanned at different wavelengths to excite the fluorescent molecules using a light source, for instance a laser. Shortly after the fluorophores have been excited they emit photons, which are registered and quantified in each position by the scanner, which results in a high-resolution digitized image for each channel. Using image analysis methods, the pixels that belong to the regions that contain the probes are identified and averaged, and an estimate of the transcript abundance for each gene is obtained.

Since these estimates are obtained from a complex measurement process of several steps, it is likely that the observed signals contain not only measurement noise, but also systematic variations of different kinds [2].

In this report, we show the existence of a channel-specific bias introduced by the scanner and most likely its detector parts. Our results indicate that the image analysis may also contribute with a small bias. The effects channel-specific biases have on the downstream microarray analysis are many [2,3]. We suggest a scan protocol and a model that will allow us to estimate the biases and calibrate the observed signals accordingly. The result will be that the intensity dependent effects are removed, but also that the effective dynamical range of the scanner is increased several times.

## Model

### General model

Consider a microarray experiment involving genes *i *= 1 ,..., *I *from RNA extracts *c *= 1 ,..., *C*. In single-channel microarrays each array measures the gene expression levels in one RNA extract, whereas in two-color microarrays each array measures two RNA extracts, one in each channel. We will refer to each set of signals from each RNA extract as *channels*. Let *μ*_*c*,*i *_be the true gene expression (transcription) level of gene *i *in channel *c*. Ideally, statistical analysis can then be done on these quantities. For instance, by comparing the relative abundances in two channels, that is *r*_*i *_= *μ*_1,*i*_/*μ*_2,*i *_for all genes *i*, it is possible to identify genes that are significantly differentially expressed (*r*_*i *_≠ 1). However, in reality we do not observe the true expression levels, but only the quantified spot intensities *y*_*c*,*i*_. The general relation between the observed and the true expression levels can be written as

*y*_*c*,*i *_= *f*_*c*_(*μ*_*c,i*_) + *ε*_*c,i*_,     (1)

where *f*_*c*_(·) is an unknown channel-specific function, which we refer to as the *measurement function*, that includes all steps in the microarray process. Moreover, we assume independent intensity dependent error terms *ε*_*c*,*i *_such that *E*[*ε*_*c*,*i*_] = 0. Because we want to do inference based on *μ*_*c*,*i*_, it must be possible to find the inverse of *f*_*c*_(·), which (at least in theory) is possible if it is strictly increasing.

To be able to find the form of *f*_*c*_(·), high quality calibration data from several stages along the microarray process is required. Here we will consider a simpler case. Split the overall measurement function into two parts. The first part *x*_*c*,*i *_= *g*_*c*_(*μ*_*c*,*i*_) models, in channel *c*, the amount of light from spot *i *that enters the photomultiplier tube (PMT) [4] as a function of the transcription level of clone *i*. The second part, which is studied in this report, is *y*_*c*,*i *_= *h*_*c*_(*x*_*c*,*i*_) and models the observed signal as a function of the amount of photons in channel *c *and spot *i *that enters the PMT. That is, it captures the characteristics of the scanner's light detector, but also of the image analysis methods. We want to emphasize that the light from one spot does not necessarily originate solely from the fluorescent molecules that are attached to the hybridized target DNA. Light from other sources such as cross-hybridized target, intrinsic fluorescence from spot buffer, and scatter light may also contribute with photons of similar wavelengths.

Next we will show that *h*_*c*_(·) is almost perfectly affine. This measurement function also depends on the scanner settings, especially the scanner sensitivity, which is indicated below with the super index (*k*). In other words,


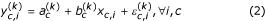


where for each fix scanner setting *k*, 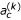 and 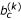 are channel-specific bias and scale parameters, respectively. Assume that *x*_*c*,*i *_is *fix *for all PMT voltages.

Note that the above relationship is not necessarily linear. Here we refer to linear in the strict sense where *a*_*c *_= 0 so that the output is proportional to the input. Lack of linearity has important implications on downstream analysis. For instance, when spotted as well as in-situ synthesized microarrays are used it is common to do statistical analysis on the log-ratios *M*_*i *_= log_2_(*y*_*R*,*i*_/*y*_*G*,*i*_) and the log-intensities 
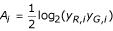
 for all genes *i *[5], where we for convenience have denoted the two channels to be compared by *R *and *G *although such comparisons are not limited to within-array measurements. One of the rationales for this bijective transform is that under ideal conditions the main measure of interest, the fold change, is contained in one variable only. However, a channel specific bias introduced by *f*_*c*_(·) will introduce a so called intensity dependent bias in the observed log-ratios. Commonly observed intensity dependent effects in the log-ratios [6] can partly be explained by the fact that the logarithm is taken on affinely transformed signals [2,3,7].

### Constrained model

**Figure 1:**
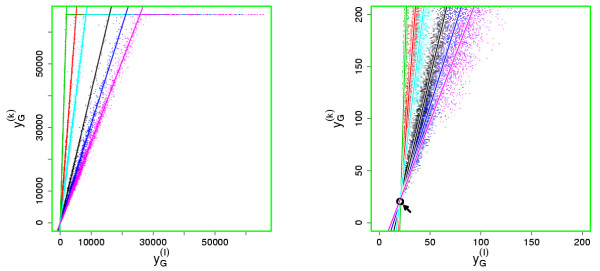
**Affine relationship between quantified fluorescent intensities and concentration of fluorophores. **Scanning the arrays at different PMT gains indicates that there is an affine relationship between quantified fluorescent intensities and concentration of fluorophores. Left: Observed signals in the green channel for PMT pairs (800, 500), (700, 500), (800, 600), (600, 500), (700, 600), and (800, 700) are shown in green, red, cyan, black, blue and magenta, respectively. An affine model is estimated for each pair and displayed as a line. Data points at the very top are saturated and ignored. Right: A zoom-in of the same data. For *each *pair the estimated 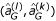, which corresponds to the true origin, has been highlighted with a circle. All lines seem to intersect at the same point on the 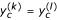 line. Shown are signals from the green channel on Array A quantified by GenePix from Axon scanner images.

The model in equation (2) is not identifiable. We address this problem as follows. Consider the case where the same array has been scanned at *K *different PMT settings. Let 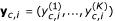 be the vector of the *K *quantified signals for gene *i *and channel *c*. In the noise-free case it follows from (2) that 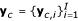 lies on the line *L*(**a**_*c*_, **b**_*c*_) in 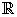
^*K*^, which has direction 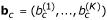 and goes through the point 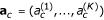. The 2*K *parameters of **a**_*c *_and **b**_*c *_are not identifiable, since *L *has only 2*K *- 2 degrees of freedom. In fact, any transformation **b**_*c *_← *k*·**b**_*c *_and **a**_*c *_← **a**_*c *_+ *l*·**b**_*c*_, where *k *and *l *are scalars, will leave *L *intact. In this paper, we make **a**_*c *_and **b**_*c *_identifiable by choosing *k *and *l *so that 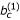 = 1 and **a**_*c *_is the point on *L *closest to the (diagonal) line *L*' = {*e*_*c*_(1,..., 1); *e_c_*∈ 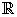}. The choice of **a**_*c *_can be motivated by looking at observed data. By inspection, we observe that the bias 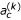 in Model (2) is not varying much when the PMT gain is changed. To demonstrate this, 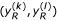 have been plotted for each of the six possible PMT pairs in Figure 1. First, the close fit of the lines to data is evidence that the scanner is linear in its dynamical range. Second, all lines go through approximately the same point, lets call it (*e*_*c*_, *e*_*c*_), suggesting that there is a common PMT-independent bias *e*_*c*_. More precisely, we split the bias term into two parts, one dependent and one independent on the PMT gain according to


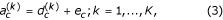


and define 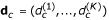 ∈ 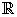
^*K*^. For this split, data indicates that ||**d**_*c*_|| ≈ 0, where ||·|| is the norm in, say, *L*_2 _(Euclidean distance). Let **d **= **y **- *e*(1,..., 1) where **y **∈ *L *and *e *∈ 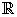. The constraint that **a**_*c *_is the point of *L *closest to *L*' can then be formulated as


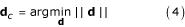


where the minimization is with respect to **y **and *e*. Equivalently, this means that **d**_*c *_is orthogonal to **b**_*c *_and (1,..., 1). The above can be interpreted geometrically as follows. By definition, **a**_*c *_is a point on the line *L*(**a**_*c*_, **b**_*c*_). Similarly, **e**_*c *_= *e*_*c*_(1,..., 1) is a point on the diagonal line that goes through (0,..., 0) and (1,..., 1) in 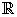
^*K*^, i.e. *L*'. Minimizing **d**_*c *_according to (4) is the same as finding the shortest distance between the line *L *and the diagonal line, which is also the distance between the two points **a**_*c *_and **e**_*c*_. From this geometrical interpretation it is also clear that in order for the parameters to be uniquely identifiable the line *L must not *be parallel to the diagonal line, that is, 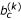 must be different from 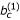 for some *k*.

A robust estimate of *L *was proposed in [2], using iteratively re-weighted principal component analysis (IWPCA). This estimate of *L*, together with the above parametrization of **a**_*c *_and **b**_*c*_, give us estimates 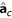 and 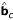 of all 2*K *- 2 parameters of **a**_*c *_and **b**_*c*_, as well as an estimate 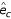 of *e*_*c*_.

Let us illustrate the parametrization and estimation procedure for *K *= 2. Since two (non-parallel) lines will always intersect, constraint (4) degenerates to the assumption that **d**_*c *_= **0 **or, equivalently, that 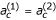 = *e*_*c *_In the noise-free case the line *L *is described by


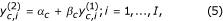


where 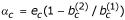 and 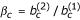. By setting 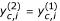 in (5) and applying the constraint 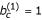, we get that **a**_*c *_= (*e*_*c*_, *e*_*c*_) and **b**_*c *_= (1, *β*_*c*_) where *e*_*c *_= *α*_*c*_/(1 - *β*_*c*_). To further illustrate the stability of the PMT independency, the parameters (*e*_*c*_, *β*_*c*_) have been estimated for each of the six PMT pairs *independently *based on data from array A scanned by the Axon scanner and quantified by GenePix. The various estimates for both channels are listed in Table 1. The average estimate of the bias across all PMT pairs in the red channel was 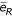 = 18.0 (with standard deviation 1.12). For the green channel the average bias estimate was 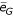 = 20.3 (with standard deviation 0.80). The small standard deviations confirm that **d**_*c *_is indeed small.

## Results

This analysis was done with eight arrays (A-H) that were scanned on two different scanners at four different PMT settings. Two different image analysis applications were used. See Methods for details.

### Parameter estimates

For every combination of array, scanner and signal quantification method (image analysis or raw pixel intensities), we estimated the parameters **a**_*c *_(including *e*_*c *_and **d**_*c*_), and **b**_*c *_in Model (2)-(4) for both channels (see Methods). To get a better understanding of the properties of the estimates, we used a non-parametric bootstrap approach to obtain not only bias corrected estimates, but also their standard deviations. Data was resampled over gene index in a way such that the same bootstrap data sets were used whenever pairwise comparison was done, e.g. when comparing bias estimates in red and green channels. For GenePix and Spot quantified signals a bootstrap sample of size 100 was used.

For the estimates based on the raw pixel intensities a different approach was taken. Because the number of pixels for one scan is about 10^7 ^(per channel) and we had four scans, our computer system limited us to estimate the model based on a subset of 10^6 ^pixel intensities. This was done for 100 random subsets and the mean and standard deviation of the parameter estimates were calculated, much like the bootstrap method above. The mean and the standard deviation of 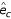 and 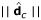 for all possible setups are listed in Tables 2 &3. The mean and standard deviation of 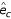 over all arrays are shown in Table 4.

#### Comparison of arrays

**Figure 2:**
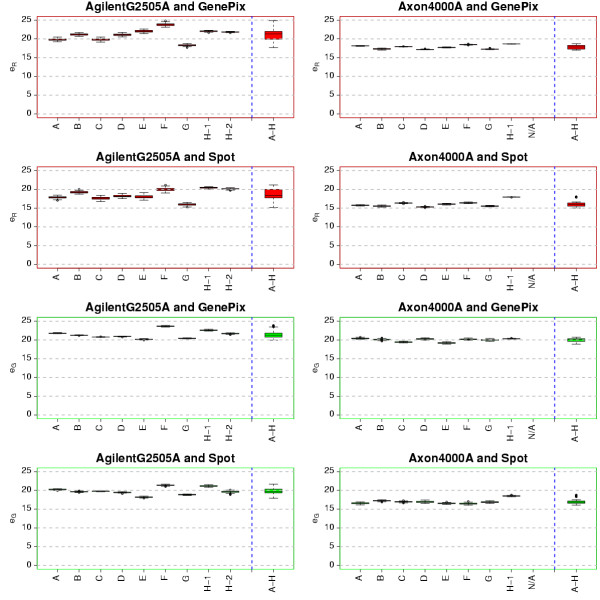
**Bias estimated for each array and channel on both scanners **Estimated biases *e*_*c *_for each array and for all arrays together from the Agilent scanner (left column) and the Axon scanner (right column). The top four graphs are for the red channel and the bottom four are for the green channel. For each channel the top two are estimates based on signals quantified by GenePix and the bottom two on signals quantified by Spot. See also Table 2 & 3.

The bias estimates for all bootstrap replicates in Tables 2 &3 have been depicted as box plots in Figure 2. Considered that the signals are in [0, 65535], the bias estimates are very stable between different arrays. The biases span 9.8 units (0.15‰) in the red channel and 7.8 units (0.12‰) in the green channel.

#### Comparison of scanners

**Figure 3:**
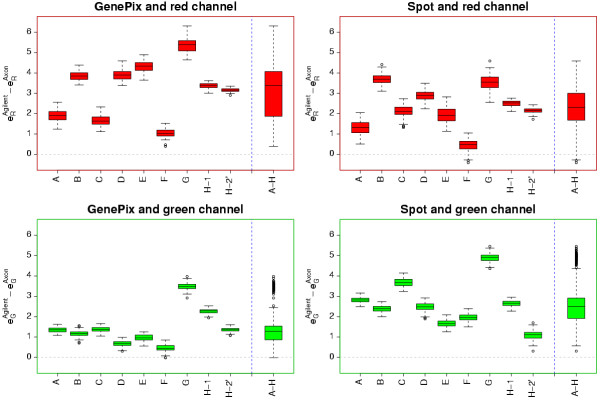
**Comparison of bias estimates between scanners. **Differences in the common biases *e*_*c *_between the Agilent and the Axon scanners for each separate array and for all arrays together. The bias is significantly larger for Agilent, regardless of channel (top and bottom) and image analysis method (left and right).

For the two scanners, we found that the estimated bias based on signals obtained by the Agilent scanner are consequently higher than the estimates from the Axon scanner. The box plots of their differences in the common bias *e*_*c *_(for each bootstrap sample) between the Agilent and the Axon scanner in Figure 3 confirm this divergence. See also Table 4. This significant difference could be an effect of scan order, that is, all arrays were first scanned on the Agilent scanner and then on the Axon scanner. The arrays in hand were part of a much bigger project based solely on Agilent scanned data. To keep a consistent scan protocol and to minimize bleaching, we could not balance the experimental design by letting some arrays be scanned in the reverse order. Instead, to test for scan order trends we scanned one array first on Agilent (H-1), then on Axon (H-1) and then again on Agilent (H-2). No apparent trend was found.

#### Comparison of image analysis methods

**Figure 4:**
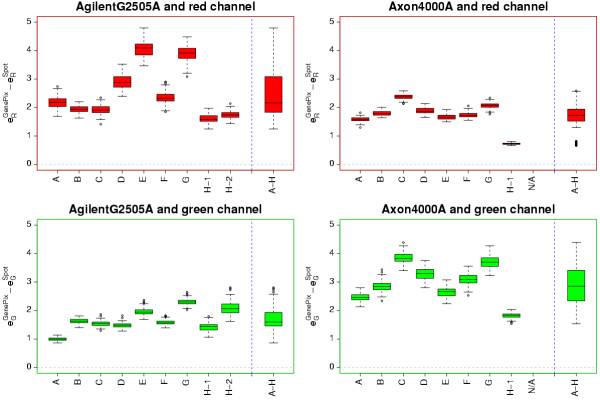
**Comparison of bias estimates between image analysis methods. **Differences in the common biases *e*_*c *_between the GenePix and the Spot image analysis method for each separate array and for all arrays together. The bias is significantly larger for GenePix, regardless of channel (top and bottom) and scanner (left and right).

Estimates of the common bias *e*_*c *_based on GenePix quantified signals are consistently greater than the corresponding ones based on Spot signals, cf. Tables 2,3,4. The box plots in Figure 4 show differences in estimates of the common bias (for each bootstrap sample) between GenePix and Spot. The difference may be explained by the fact that the two applications use different spot segmentation algorithms [8,9]. Because the concentration of fluorophores is not homogeneous across a spot, the result is that the distribution of pixel intensities will vary with the segmentation method. This effect can be more profound for spots with strong donut effects. Robust estimates such as the median pixel value will to some extent protect against this variance, but not completely. It has been suggested [10] that the *median of *(pixel) *ratios *is a better estimate of the ratio of hybridized cDNA than the *ratio of median *(pixels). However, the former requires that the images are perfectly aligned with respect to shift, rotation, shear and so on. Also, it applies exclusively to two sample comparisons. Because of this, we do not believe that pixel-ratio signals are useful in practice.

#### Pixel-based estimates

To better understand the underlying reasons for the observed channel biases, the proposed affine model was also applied to pixel intensities (instead of spot signals). The estimated biases for the two channels for different arrays using IWPCA based on *pixel values *are shown in Tables 2 &3. Except for the green channel in the second scan round of Array H, the pixel-based estimates are consistently higher than the estimates based on GenePix and the Spot foreground signals. As noted above, pixel-based estimates are very sensitive to image distortions. This is especially a concern for the Agilent scanner since it reloads the arrays between subsequent scans. To investigate the effect of image distortion, we did a test where a person with experience in microarray analysis was asked to subjectively rank how badly aligned the four images in the red channel with different PMT gains from the Agilent scanner were for each of the (unlabeled) nine arrays. The person rated the images from Arrays A, B, D, and H-1 to be "extremely" misaligned. The images from Array E were considered to be "quite" misaligned, and the images from Array C to be "slightly" misaligned. For the rest of the arrays the images were considered to be aligned (less than a pixel off). This is perfectly in line with the discrepancies in Table 2, which confirms our hypothesis. Another disadvantages with pixel-based methods is that they are extremely memory and time consuming. For instance, estimation with 10^6 ^pixels is approximately 50 times slower than with 55,488 signals.

#### Comparison of channels

**Figure 5:**
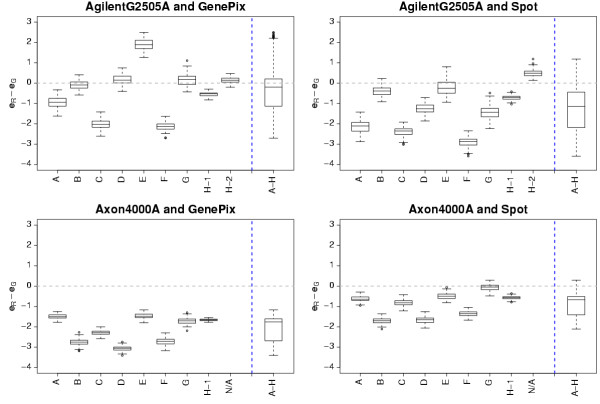
**Comparison of bias estimates between channels. **Differences in the common biases *e*_*c *_between the red and the green channel for each separate array and for all arrays together. At the top is data from the Agilent scanner and at the bottom from the Axon scanner. Data in the left column by GenePix and Data in the right column was quantified by Spot. For data from the Axon scanner the common bias is greater in the green channel than the red channel, especially for GenePix quantified signals. For the Agilent scanner this trend is less clear although the Spot quantified signals clearly seem to have a higher bias in the green than the red channel.

As Figure 5 shows, the common bias *e*_*c *_is greater in the green channel than in the red channel, especially for GenePix quantified signals, when estimated based on data from the Axon scanner. For the Agilent scanner this trend is less clear although the Spot quantified signals seem to give higher bias in the green channel than in the red channel. Furthermore, the biases in the red and the green channels are stable between arrays, which give further evidence to our hypothesis that the bias originates from the scanner (and/or the image analysis methods).

#### Deviation in bias estimates between PMT gains

**Figure 6:**
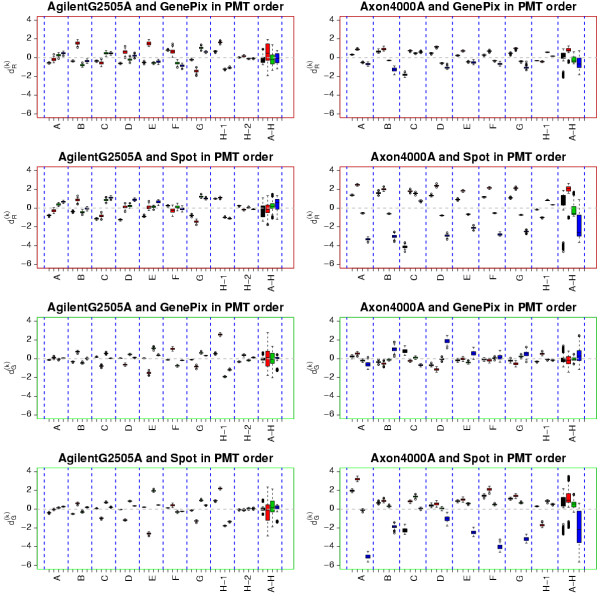
**Bias residual estimates for all arrays. **Estimated bias residuals 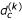 for each array and for all arrays together from the Agilent scanner (left column) and the Axon scanner (right column). For each array the distribution of the four of 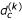 are shown in increasing *PMT order*. The top four graphs are for the red channel and the bottom four are for the green channel. For each channel the top two are estimates based on signals quantified by GenePix and the bottom two on signals quantified by Spot.

In Figure 6 the distribution of the "bias residuals" 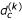 are depicted for different scans *k *and channels *c*, for each separate array, but also for all arrays together, and for both scanners and both image analysis methods. Most apparent is that the estimates based on signals from the Axon scanner and especially those quantified by the Spot software are greater than for the others, cf. Tables 2 &3. The reason for this difference is not clear to us. For some arrays the estimates from the red and the green channels are strongly correlated, but it is not clear to us when this occurs. Although not in general, for some combinations of scanner and image analysis method, there is a trend in the PMT order (or possibly scan order). Again, we do not know why. To summarize, we have by means of exploratory data analysis (not shown) tried to understand what sometimes looks like patterns in the 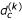 :s, but we found no apparent relationships. However, systematic effects indicate that 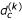 may be modeled further.

### Calibration

**Figure 7:**
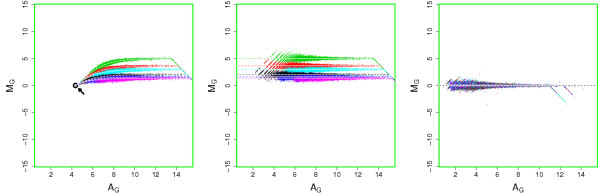
**Log-ratio and log-intensity plots of raw, translated, and calibrate signals. **The affine transformation gives curvature in the *M *versus *A *plots, which is corrected for by the affine normalization method. The three scatter plots show the within-channel log-ratio versus the log-intensity for each of the six PMT pairs based on the same data as in Figure 1. Left: Observed signal for different PMT pairs. For *each *pair the estimated 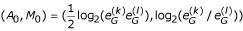 has been marked with a circle, cf. Figure 1. Mid: Bias corrected signals. Right: Bias and scale calibrated signals. The range of the *M *axis is twice the range of the *A *axis so that (6)-(7) appear as a rotation in the plots.

When data was calibrated according to the backward transformation in (8)-(10) estimates (up to a scale factor) of all *x*_*c*,*i*_:s were obtained. Since we do not know the true values we can not verify the estimates directly. However, partly we can do it indirectly by looking for remaining systematic effects in the log-ratios, but also by comparing the empirical densities of the calibrated scans. For a detailed study on systematic effects introduced by affine transformations, see [2]. For instance, the amount of intensity-dependent curvature in the log-ratios is related to the bias and the relative scale factor via the product 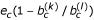 assuming ||**d**_*c*_|| = 0. To demonstrate this relationship, we have for different PMT pairs compared the within-channel log-ratios and log-intensities


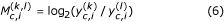



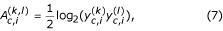


respectively, with the corresponding ones for the backward transformed data, which we denote by 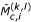 and 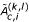. The log-ratios versus the log-intensities for the *raw signals *of all six PMT pairs are shown in the left scatter plot in Figure 7. The corresponding plot for the *backward transformed signals *is shown to the very right. For each of the six data clouds, the curvature, but also the overall bias, in the log-ratios is removed. To further underline the effect that a channel-specific *bias *has, we have calculated the log-ratios for the *bias-subtracted *signals (no rescaling), which makes Model (2) *linear*. As seen in the middle scatter plot, the curvature introduced by the bias and the logarithm is removed. The overall bias in the log-ratios which remains is 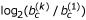 and is removed when the signals are rescaled. It is not correct to shift only the log-ratios towards zero, because then the log-intensities will be incorrect.

**Figure 8:**
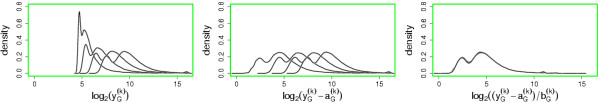
**Densities of the logarithm of the raw, translated, and calibrate signals. **The affine normalization method makes the signal densities much more similar. Left: Density plots of the logarithm of the raw signals for each of the fours scans. Mid: Bias corrected signals. Right: Bias and scale calibrated signals. Data and colors are the same as in Figure 1.

The various *M *versus *A *plots become very similar and so do the four empirical density functions of the signals as seen in Figure 8. The small bumps at high intensities are due to the saturated signals, cf. Figure 7.

#### Extended dynamical range

For the Agilent scanner the effective scale parameters 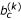/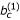 were estimated to be in the order of approximately 1 : 3.5. For the Axon scanner they were in the order of approximately 1 : 40, cf. Table 1. Thus, the calibration method extends the effective dynamical range, with preserving linearity, by a factor of 3.5 for signals from the Agilent scanner and a factor of 40 for signals from the Axon scanner.

## Discussion

### Sources of the bias

Because bias introduced before the PMT would be amplified differently at different gains, we suspect that the observed bias is due to the scanner and most likely its detector parts such as the analogue-to-digital converter (ADC) after the PMT, but possibly also due to the image analysis method. The observed differences between the channels can be explained by the fact that there is one PMT and one ADC per channel, which may have slightly different properties. Although there are differences in bias between the two scanners, they are still of the same order, which we find remarkable. Another lab with a GenePix scanner reported biases also around 15–20 (personal communication). A possible reason for this is that the scanners consist of similar parts.

### Other estimates

To rule out the obvious situation where *all *pixel intensities are biased we compared the above estimates with the *minimum pixel intensities*. For example, for Array A (scanned on Axon and analyzed with GenePix Pro), the *minimum pixel intensities *in the red channel were 9, 0, 8, and 9 for PMT 500, 600, 700 and 800 volts, respectively. In the green channel the minimum pixel intensity is 0 for all scans. It is not useful to use the *minimum spot signals*, 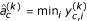, either. For example, for the above scan the average minimum signal across all scans in the red channel is 19.8 (median 19.5, std. dev. 0.96), but in the green channel it is 34.8 (median 28.0, std. dev. 19.6), cf. Table 3.

### On background subtraction

If the scanner is the main source for the observed bias, then the background estimates should be affected by this bias as well and subtracting the background from the foreground estimates will therefore not only correct for physical background noise from the array itself, but also for the scanner bias. The *strong *intensity dependent effects of the log-ratios that are due to the bias, are much less apparent if we apply background subtraction (not shown), giving more evidence to our hypothesis that the observed systematic effects originate from the scanner. Thus doing background correction might correct for the bias, but it will also introduce more noise at any given intensity. Also, for the data set in hand *background subtraction *results in 4050 (7.3%), 6237 (11%), 7015 (13%) and 7349 (13%) negative values (in either channel), respectively, whereas *bias subtraction *results in no negative values. If we assume that the noise is additive such that the background is added to the foreground signals, then for probes with few or no fluorescent molecules the true foreground signal should be close or identical to the true background signal. As both are *estimates*, approximately half of the foreground signals for non-signal spots are less or equal to the corresponding background signals. Thus, about half of such spots results in negative signals. However, the different numbers of negative signals for different PMT voltages suggest that this can not be the full explanation. One reason could be that the background estimates are likely to be biased [9]. An error model that incorporates different noise sources, but also different scan parameters, might give some answers to this problem. Some models in this context have already been suggested [3,7,11], as well as models based on empirical Bayesian methods [12]. Another way to put it is that the background estimate is local and based on individual spots/pixels whereas the bias estimate is global, that is, there is one estimate for the whole array (although local estimation of bias is possible). Therefore, the background subtracted intensity estimates are noisier, resulting in more negative estimates for low intensity spots.

The problem of non-positive estimates, but also high variance close to zero, are limitations of the logarithmic transform and alternatives such as the generalized logarithmic transform etc. have been suggested [7,13,14].

### Photo bleaching

We estimated the red dye (Cy5™) to bleach about 2% and the green dye (Cy3™) about 1% in a typical microarray experiment (not shown). Because the amount of bleaching is relatively small, but also because it is a very complex phenomenon, we decided to not try to incorporate it in the above model. Some of the systematic variation seen in the bias estimates for the different PMT settings may be due to bleaching.

### Signal density normalization

As the results show, the empirical distributions of signals match each other remarkably well after calibration. It is interesting to compare this method with the quantile normalization methods proposed by [15-17]. The latter is based on the "statistical" assumption that the signals in all channels (scans) *should be equal *whereas the former is based on a "physical" assumption that the signals *should be linear *in the dynamical range. For a further discussion on this see [2].

### Incremental robust estimates

It turned out to be infeasible to estimate the model parameters based on all *pixel intensities*, which limited us to use only on a 10% subset of data. As argued above, pixel-based estimates are not reliable and therefore not of interest. However, for spot-based estimates the same limitations may apply as larger data sets are made available. We wish to overcome such memory constraints. For this reason, we investigate the possibility to use (approximative) incremental re-weighted PCA methods [18,19].

### Related work

Another method that combines multiple scans is the *masliner *(Microarray Spot LINEar Regression) algorithm [20]. It works by combining one low-PMT scan and one high-PMT scan into a new virtual scan. If a signal in the high-PMT scan is within a specified linear range its value is used, otherwise the corresponding signal from the low-intensity range is used after being transformed affinely to fit the high-PMT scan. To combine three or more scans, the new virtual scan can be combined with another PMT scan and so on. The result is that the effective dynamical range is extended. However, there are several unnecessary drawbacks. First, although several observations of the same spot concentration exist, which all may be within the dynamical range of the scanner, only one observation is used. Statistically, the average (calibrated) scan would be a more precise estimate. Second, since the scans are combined pairwise the estimate of the affine relationship between the scans is less robust. Third, although a sensitivity discussion is carried out in the supplementary materials, masliner fits the affine models in a non-robust fashion (in *L*_2_). Also, classical linear regression is used, which assumes no error in the explanatory variable. Since masliner makes the signals from different PMT settings *proportional to each other *it will indeed remove for instance curvature in within-channel *M *versus *A *scatter plots. However, masliner does not model the possibility of a PMT-independent bias and will therefore not correct for it. We believe this is the reason why the authors observe a "curvilinear effect" [[20], supplementary material]. For these reasons, we believe that the robust multiscan calibration method presented in this paper is superior to the masliner algorithm and should be used instead.

## Conclusions

By scanning the same microarray at various PMT settings we have shown that there exists a bias in the measurement of the concentration of fluorescent molecules in the spots on the microarray. Our analysis indicates that this bias is mainly due to the scanner, but also due to the image analysis methods. By using a constrained affine model for the relationship between the obtained fluorescent intensities and fluorophore concentrations in the spots, we have been able to estimate the aforementioned bias. With estimates of the bias and scale parameters in each channel back transformation gave estimates of the amount (up to a scale factor) of photons from each spot that enters the PMT. Although not all photons originate solely from fluorophores in the target DNA, this is still a far better estimate of the amount of hybridized target DNA in each spot than the corresponding signal quantified by the scanner and the image analysis.

Before calibration, our data show a strong intensity dependent effect in the log-ratios, whereas after calibration there is no apparent intensity dependent trend. Furthermore, the distributions of signals from subsequent PMT scans are almost identical after calibration. In addition, the signal-to-noise ratio is increased with multiple scans. Finally, scanning at both low and high PMT settings extends the dynamical range of data, which gives higher resolution at low intensities without having to pay the price of saturated signals.

The proposed method can be applied to other microarray technologies such as single-channel oligonucleotide arrays or nylon arrays, and possibly to other gene expression technologies such as quantitative real-time polymerase chain reaction (QRT-PCR).

To conclude, we suggest that hybridized microarrays are scanned at two (preferably more) PMT gain levels to identify channel dependent bias terms. Knowing the exact PMT settings is not important, but the larger the differences are, the more precise the estimates will be. We recommend that the scans are done in *decreasing *PMT-gain order (although we did not do so here). Given estimates, data can then be calibrated easily. For practical reasons it might, however, be sufficient to estimate bias terms for a specific scanner once and then use estimates for calibration of subsequent microarrays. The small inter-array variation observed for channel specific bias in our data suggests that this would be possible. On the other hand, without multiple scans, afore mentioned increase in signal-to-noise and dynamical range will be lost. Also, not investigated within the scope of this study, bias terms for a specific scanner might change over time. For these reasons, we suggest that microarrays are scanned multiple times.

For two-channel microarrays, after calibrating each channel separately, a similar strategy can be applied once more to bring differently labeled channels to the same scale as suggested in [2]. This would rely on the assumption that the amounts of hybridized DNA in all channels are approximately equal for the majority of the spots, which in turn is based on the commonly used assumption that most genes are non-differentially expressed. This also applies to normalization between arrays.

All necessary methods are made available in a free R package named aroma [21]. A typical usage is calibrateMultiscan(rg) where rg is the object containing the red and green signals. In addition, we are currently implementing the methods as a plug-in module for the BASE system [22].

## Methods

### Arrays and hybridization

The analysis was based on eight different hybridizations of spotted oligonucleotide microarrays (A-H). Arrays A and B were hybridized in October 2003. Arrays C-G were hybridized the following day and Array H was hybridized seven weeks later. All arrays contain the same human oligonucleotide set (QIA GEN) and all have an identical layout of 12-by-4 print-tip groups each containing 34-by-34 (1156) spots. In total there are 55488 spots on each array. The average (GenePix) spot area is 45–50 pixels and the average center-to-center distance between the spots is approximately 12–13 pixels (120–130 *μm*). Arrays were produced by the SWEGENE DNA Microarray Resource Centre, Department of Oncology at Lund University using a MicroGrid II 600R arrayer fitted with MicroSpot 10 K pins (BioRobotics). All arrays except Array H were spotted in the same print batch on UltraGAPS™ coated slides (Corning Incorporated) during August 2003. Array H was spotted in October the same year. Printing was performed in a temperature (18–20°C) and humidity (44–49% RH) controlled area. After printing was completed, arrays were left in a desiccator to dry for 48 hours, rehydrated for 1 second over steaming water, snap dried on a hot plate (98°C), UV-cross-linked (800 mJ/cm^2^) and subsequently hybridized with various test and reference RNA samples. Samples were labeled, purified and hybridized using Pronto!™ Plus System 6 (Corning Incorporated) according to manufacturer's instructions.

### Scanning

Each array was scanned at four different PMT settings on two different types of scanners. First the arrays were scanned on an Agilent G2505A DNA microarray scanner (Agilent Technologies) at PMT gains 100%, 30%, 50%, and 80% (in that order). The so called *dark offset *intentionally added to all signals by the Agilent scanner [[23], p. 18] has been uninstalled. Arrays were then re-scanned on an Axon GenePix 4000 A scanner (Axon Instruments) at PMT gains 600, 700, 800, and 500 volts (in that order), except for Array A, which was scanned at 700, 800, 500 and 600 volts, and Array H, which was scanned at 600, 400, 500 and 700 volts. Thus, the images obtained by the Axon scanner were bleached more than the preceding ones obtained by the Agilent scanner. For both scanners the power of the 532 nm and the 635 nm lasers was set to 100% and the scan resolution to 10 *μm*/pixel. Moreover, a one-pass (both channels scanned simultaneously) and one-sample-per-pixel ("lines to average" equals one) procedure was used. The Agilent scanner has a special loading mechanism for microarrays, which allows automatic scanning of subsequent arrays without human intervention. However, due to limitations in the software or the scanner, each batch of arrays can only be scanned at a single PMT gain. To scan at more PMT gains with the Agilent scanner, it was therefore necessary to eject and reload the arrays between different settings, which means that the alignment between the scanned images may not be perfect. Contrary, for the Axon scanner the arrays were put in the scanner one by one, then scanned at all PMT settings without being moved.

### Image analysis – spot segmentation and registration

To quantify the foreground and the background signals, the scanned images (65536 gray scales and approximately 2000-by-5600 pixels) were analyzed using both the Axon GenePix Pro v4.1.1 software (Axon Instruments) and the Spot v2 software [8,24]. We first analyzed each image with GenePix. For each of them, the grid and spot positions were manually set and then the alignment was optimized by GenePix. These positions were then re-entered and re-optimized by Spot with visual inspection to verify the correctness. Moreover, for each individual scan the image analysis software was let to find the optimal spot segmentation. Thus, what is defined as a foreground pixel may vary with PMT setting although the images are from the same array. We decided on this schema for various reasons. The first reason was that the Agilent arrays are loaded and unloaded between subsequent scans and therefore require a separate spot segmentation. To be able to compare the results from the Axon and the Agilent scanner we choose the same procedure for the images scanned on the Axon scanner, even though, the optimized segmentation for the strongest image could have been reused. We further believe that this allows us compare Spot and GenePix more fairly.

For both Spot and GenePix the median spot pixel intensity was used as foreground signal. Background estimates were not considered in this analysis. No spot signals were discarded.

### Calibration

Given estimates of 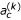 and 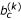 data can be calibrated using backward transformation. Let


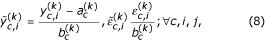


be the backward transformed observed signal and the rescaled error terms, respectively. The affine Model (2) can then be rewritten as


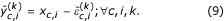


Moreover, let


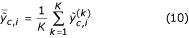


be the average backward transformed signal for gene *i *in channel *c*. Now, if 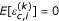, then


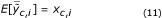


when all 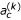 and 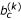 are known. Thus, if (8) is applied with estimates of 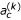 and 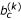 that are consistent as *I *→ ∞, and the error terms have zero mean, the mean of the backward transformed signals will converge to *x*_*c*,*i *_as *I *grows. Even though 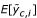 is not observable, we can estimate it consistently by increasing the number of scans *K*. Inspection of the residuals of calibrated signals (not shown) indicates that the variance of the calibrated noise is independent of PMT setting, that is 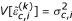. Assuming independent noise terms, the variance of the sample mean decrease with *K *as


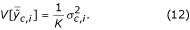


In summary, we obtain consistent estimates (up to a multiplicative constant) of all *x*_*c*,*i *_with increasing *I *and *K*.

Finally, signals that are saturated by the scanner have to be excluded before calculating the average. If the quantified signal for a spot happens to be saturated in all scans, then that spot is marked as saturated, which still may be informative when compared to other non-saturated signals.

### Data analysis

All further analysis was carried out using R [25,26] and the aroma package (f.k.a. com.braju.sma) [21]. All methods used can be found in the latter.

## Authors' contributions

GJ and JVC carried out the practical microarray laboratory work and the scanning of hybridized arrays. Image analysis using GenePix software was carried out by JVC whereas HB carried out image analysis using Spot software. HB performed all the statistical analysis and conceived the constrained affine model used to identify and estimate channel-specific bias in microarray data. All authors participated in the design of the study and approved the final manuscript.

## Figures and Tables

**Table 1:** Pairwise parameter estimates. Red and green channel estimates of the bias and the slope for each PMT voltage pair together with the mean and the standard deviation of all estimates. The small standard deviation is evidence that the bias to a large extent is independent of the PMT settings. Data shown originates from Array A scanned on Axon and analyzed with GenePix.

PMT pair	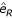	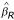	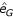	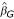
(800, 500)	18.9	32.4	20.7	32.5
(700, 500)	19.0	13.0	20.9	12.8
(800, 600)	17.3	7.1	19.8	7.9
(600, 500)	19.0	4.5	20.8	4.1
(700, 600)	17.6	2.9	20.9	3.1
(800, 700)	16.3	2.5	18.9	2.6
mean	18.0 ± 1.12		20.3 ± 0.80	

**Table 2:** Red channel parameters estimates. Bootstrapped parameter estimates for the red channel with standard deviations for each combination of array, image method (or pixel intensities) and scanner.

array	image	Agilent		Axon	
	method	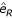	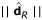	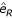	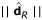
A	Spot	17.9 ± 0.289	1.14 ± 0.148	15.8 ± 0.090	4.40 ± 0.145
A	GenePix	19.8 ± 0.253	0.82 ± 0.162	18.1 ± 0.058	1.27 ± 0.095
A	pixels	44.9 ± 0.036	22.85 ± 0.043	19.0 ± 0.032	0.68 ± 0.052
B	Spot	19.2 ± 0.255	1.05 ± 0.165	15.5 ± 0.121	4.01 ± 0.255
B	GenePix	21.2 ± 0.225	1.83 ± 0.203	17.3 ± 0.114	1.72 ± 0.253
B	pixels	42.8 ± 0.049	20.71 ± 0.051	17.8 ± 0.034	2.06 ± 0.079
C	Spot	17.6 ± 0.366	2.00 ± 0.192	16.3 ± 0.076	4.83 ± 0.191
C	GenePix	19.8 ± 0.284	0.94 ± 0.209	17.9 ± 0.034	2.11 ± 0.135
C	pixels	21.0 ± 0.138	1.48 ± 0.079	18.8 ± 0.022	1.24 ± 0.061
D	Spot	18.2 ± 0.301	1.56 ± 0.103	15.3 ± 0.093	4.10 ± 0.149
D	GenePix	21.1 ± 0.274	0.95 ± 0.139	17.2 ± 0.049	1.74 ± 0.090
D	pixels	25.0 ± 0.522	4.10 ± 0.358	18.3 ± 0.025	1.02 ± 0.044
E	Spot	18.0 ± 0.428	1.15 ± 0.109	16.1 ± 0.101	3.01 ± 0.131
E	GenePix	22.1 ± 0.268	1.77 ± 0.223	17.7 ± 0.064	1.02 ± 0.096
E	pixels	24.7 ± 0.144	5.51 ± 0.169	18.7 ± 0.024	0.39 ± 0.025
F	Spot	19.9 ± 0.423	0.48 ± 0.163	16.4 ± 0.087	3.76 ± 0.138
F	GenePix	23.8 ± 0.316	1.47 ± 0.258	18.5 ± 0.060	1.07 ± 0.106
F	pixels	24.2 ± 0.131	1.37 ± 0.101	19.3 ± 0.026	0.43 ± 0.038
G	Spot	16.0 ± 0.300	2.30 ± 0.166	15.5 ± 0.077	3.54 ± 0.114
G	GenePix	18.3 ± 0.208	1.88 ± 0.198	17.3 ± 0.070	1.57 ± 0.134
G	pixels	19.1 ± 0.096	1.48 ± 0.080	18.3 ± 0.026	0.66 ± 0.038
H-1	Spot	20.4 ± 0.161	2.12 ± 0.073	17.9 ± 0.025	1.35 ± 0.043
H-1	GenePix	22.1 ± 0.124	2.41 ± 0.097	18.7 ± 0.024	0.81 ± 0.041
H-1	pixels	44.9 ± 0.513	17.68 ± 0.528	19.1 ± 0.013	0.78 ± 0.027
H-2	Spot	20.1 ± 0.141	0.33 ± 0.040	N/A	N/A
H-2	GenePix	21.8 ± 0.087	0.22 ± 0.084	N/A	N/A
H-2	pixels	21.8 ± 0.047	0.26 ± 0.042	N/A	N/A

**Table 3:** Green channel parameters estimates. Bootstrapped parameter estimates for the green channel with standard deviations for each combination of array, image method (or pixel intensities) and scanner.

array	image	Agilent		Axon	
	method	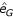	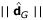	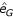	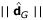
A	Spot	20.2 ± 0.106	0.51 ± 0.058	16.6 ± 0.165	6.31 ± 0.302
A	GenePix	21.8 ± 0.068	0.20 ± 0.045	20.4 ± 0.123	0.88 ± 0.242
A	pixels	33.6 ± 0.050	10.52 ± 0.048	22.8 ± 0.048	1.57 ± 0.093
B	Spot	19.6 ± 0.094	0.90 ± 0.055	17.2 ± 0.127	2.20 ± 0.231
B	GenePix	21.3 ± 0.046	0.94 ± 0.086	20.1 ± 0.152	1.20 ± 0.326
B	pixels	35.7 ± 0.153	12.85 ± 0.146	21.9 ± 0.041	2.04 ± 0.087
C	Spot	19.8 ± 0.050	1.21 ± 0.073	17.0 ± 0.127	2.75 ± 0.254
C	GenePix	20.8 ± 0.039	0.99 ± 0.086	19.4 ± 0.100	1.11 ± 0.218
C	pixels	21.3 ± 0.024	0.78 ± 0.035	22.0 ± 0.040	2.39 ± 0.082
D	Spot	19.4 ± 0.097	1.49 ± 0.073	17.0 ± 0.194	1.24 ± 0.376
D	GenePix	20.9 ± 0.059	0.80 ± 0.095	20.3 ± 0.126	2.31 ± 0.286
D	pixels	21.6 ± 0.105	0.21 ± 0.124	22.1 ± 0.046	3.10 ± 0.092
E	Spot	18.2 ± 0.121	3.29 ± 0.129	16.5 ± 0.132	2.88 ± 0.240
E	GenePix	20.2 ± 0.081	1.92 ± 0.128	19.2 ± 0.137	0.74 ± 0.264
E	pixels	21.2 ± 0.017	0.13 ± 0.046	21.0 ± 0.032	0.94 ± 0.058
F	Spot	21.4 ± 0.125	0.58 ± 0.103	16.5 ± 0.181	4.79 ± 0.327
F	GenePix	23.7 ± 0.084	1.32 ± 0.116	20.2 ± 0.132	0.34 ± 0.199
F	pixels	24.1 ± 0.019	1.57 ± 0.026	22.4 ± 0.039	1.87 ± 0.071
G	Spot	18.9 ± 0.088	1.62 ± 0.088	16.9 ± 0.149	3.72 ± 0.258
G	GenePix	20.4 ± 0.049	1.10 ± 0.088	20.0 ± 0.164	0.84 ± 0.262
G	pixels	21.0 ± 0.017	0.75 ± 0.024	22.2 ± 0.045	2.60 ± 0.085
H-1	Spot	21.2 ± 0.134	3.25 ± 0.080	18.5 ± 0.090	2.00 ± 0.170
H-1	GenePix	22.6 ± 0.100	3.47 ± 0.107	20.3 ± 0.043	0.63 ± 0.100
H-1	pixels	42.8 ± 1.417	16.68 ± 1.843	21.3 ± 0.033	1.82 ± 0.070
H-2	Spot	19.6 ± 0.229	0.16 ± 0.077	N/A	N/A
H-2	GenePix	21.7 ± 0.104	0.55 ± 0.076	N/A	N/A
H-2	pixels	21.5 ± 0.086	0.29 ± 0.021	N/A	N/A

**Table 4:** Mean and standard deviation of bias estimates. Mean and standard deviation of the bias estimates of all arrays and for each signal quantification method. The median and MAD (median absolute deviation) of ditto gives very similar results expect for Agilent's pixel estimates, which become smaller but are still greater than the others.

image	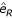		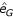	
method	Agilent	Axon	Agilent	Axon
Spot	18.6 ± 1.40	16.1 ± 0.792	19.8 ± 0.958	17.0 ± 0.633
GenePix	21.1 ± 1.55	17.8 ± 0.529	21.5 ± 1.05	20.0 ± 0.433
pixels	29.8 ± 10.4	18.7 ± 0.446	27.0 ± 7.76	22.0 ± 0.537
